# Dualsteric modulators of the M2 muscarinic acetylcholine receptor

**DOI:** 10.1186/1758-2946-6-S1-P40

**Published:** 2014-03-11

**Authors:** Marcel Bermudez, Gerhard Wolber

**Affiliations:** 1 Computer-Aided Drug Design, Institute of Pharmacy, Freie Universität Berlin, 14195 Berlin

## 

G-protein coupled receptors (GPCRs) trigger multiple signal-switching mechanisms like binding of ß-arrestin proteins, activation of kinases and G-protein activation [1]. The poor understanding of the conformational changes resulting in these activations is a major challenge for the design of specific GPCR modulating drugs. For the muscarinic M2 receptor, allosteric, orthosteric and dualsteric binding small molecules are available, which helps to elucidate multiple signaling roles [2-4]. The recently published crystal structure of the M2 muscarinic acetylcholine receptor (PDB: 3UON [5]) and mutational studies offer the possibility to rationalize and understand the binding of ligands to muscarinic acetylcholine receptors.

We present the results of extensive molecular dynamics simulations in combination with docking and 3D-pharmacophore analyses of known ligands (atropine and scopolamine) and their related dualsteric hybrid structures (JSW253, JSW257, JSW254 and JSW256). Insights into the flexibility of the allosteric binding pocket confirm earlier hypotheses: A comparison of dualsteric hybrid structures proves the crucial role of the tropane ring system for the arrangement of the allosteric part of the ligands. Whereas the extracellular loop 2 is engaged in the binding of the scopolamine-based hybrid structures, it plays a minor role for the binding of atropine-based dualsteric ligands. Orthosteric ligand binding was similar for all ligands and characterized by an essential electrostatic interaction and an aromatic cage.
